# *MRPS23* amplification and gene expression in breast cancer; association with proliferation and the non-basal subtypes

**DOI:** 10.1007/s10549-020-05532-6

**Published:** 2020-01-16

**Authors:** Elise Klæstad, Signe Opdahl, Monica Jernberg Engstrøm, Borgny Ytterhus, Elisabeth Wik, Anna Mary Bofin, Marit Valla

**Affiliations:** 1 Department of Clinical and Molecular Medicine, Faculty of Medicine and Health Sciences, Norwegian University of Science and Technology, Erling Skjalgssons gate, 7030 Trondheim, Norway; 2 Department of Public Health and Nursing, Faculty of Medicine and Health Sciences, Norwegian University of Science and Technology, Trondheim, Norway; 3 Department of Breast and Endocrine Surgery, St. Olav’s Hospital, Trondheim University Hospital, 7006 Trondheim, Norway; 4 Department of Clinical Medicine, Section for Pathology, Centre for Cancer Biomarkers CCBIO, University of Bergen, 5021 Bergen, Norway; 5 Department of Pathology, Haukeland University Hospital, 5021 Bergen, Norway; 6 Department of Pathology, St. Olav’s Hospital, Trondheim University Hospital, 7006 Trondheim, Norway

**Keywords:** *MRPS23*, Breast cancer, Proliferation, Copy number, Amplification, METABRIC

## Abstract

**Purpose:**

*MRPS23* is recognized as a driver of proliferation in luminal breast cancer. The aims of the present study were to describe *MRPS23* copy number change in breast cancer, and to assess associations between *MRPS23* copy number change and molecular subtype, proliferation and prognosis, and between *MRPS23* gene expression and molecular subtype and prognosis.

**Methods:**

Using fluorescence in situ hybridization (FISH), we examined *MRPS23* and centromere 17 copy number in 590 formalin-fixed, paraffin-embedded primary tumours and 144 corresponding lymph node metastases from a cohort of Norwegian breast cancer patients. Furthermore, we analysed *MRPS23* gene expression data in 1971 primary breast cancer tumours from the METABRIC dataset. We used Pearson’s *χ*^2^ test to assess associations between *MRPS23* copy number and molecular subtype and proliferation, and between *MRPS23* expression and molecular subtype. We studied prognosis by estimating hazard ratios and cumulative incidence of death from breast cancer according to *MRPS23* copy number and *MRPS23* expression status.

**Results:**

We found *MRPS23* amplification (mean *MRPS23* copy number ≥ 6 and/or *MRPS23*/chromosome 17 ratio ≥ 2) in 8% of primary tumours. Copy number increase associated with non-basal subtypes and higher tumour cell proliferation (Ki67). Higher *MRPS23* expression associated with the Luminal B subtype. We found no significant association between *MRPS23* amplification or *MRSP23* gene expression, and prognosis.

**Conclusion:**

Amplification of *MRPS23* is associated with higher proliferation and non-basal subtypes in breast cancer. High *MRPS23* expression is associated with the Luminal B subtype.

**Electronic supplementary material:**

The online version of this article (10.1007/s10549-020-05532-6) contains supplementary material, which is available to authorized users.

## Introduction

Increased proliferation is a hallmark of cancer [1, 2], and identification of genetic drivers of proliferation could be important for prognostication and development of new targeted treatment. By high-throughput genomic analyses, Gatza et al. identified proliferation driving genes in non-basal breast cancer [3]. Amplification of four of these genes (*MRPS23*, *FGD5*, *DTX3* and *METTL6*) was associated with a poor prognosis. Mitochondrial ribosomal protein S23 (*MRPS23*) is located on the long arm of chromosome 17 (17q22) and belongs to the mitochondrial ribosomal protein gene family [4, 5]. Mitochondrial ribosomes are composed of a small 28S subunit and a large 39S subunit. *MRPS23* encodes the 28S subunit [4, 5]. High *MRPS23* expression has been found in colon [6], cervical [7, 8] and hepatocellular cancer [9, 10], and associated with poor prognosis in non-basal breast cancer [3], hepatocellular [10] and cervical cancer [7, 8]. In a breast cancer mouse model, *MRPS23* knock-down reduced proliferation, induced apoptosis and limited angiogenesis and lymph node metastasis [11].

Our group has previously reclassified breast cancer tumours from a large cohort of Norwegian women into six molecular subtypes based on immunohistochemistry (IHC) and chromogenic in situ hybridization (CISH) [12]. The aims of the present study were to characterize *MRPS23* copy number alterations by fluorescence in situ hybridization (FISH) on formalin-fixed, paraffin-embedded (FFPE) primary tumour tissue and corresponding lymph node metastases from this cohort, and to assess how these copy number alterations associates with molecular subtypes, proliferation and prognosis. Furthermore, using the METABRIC dataset [13], we assess how *MRPS23* gene expression levels correlate with molecular subtypes and prognosis.

## Materials and methods

### Study populations and specimen characteristics

#### Cohort 1

Between 1956 and 1959, 25,727 women born 1886–1928 were invited to attend a clinical examination for early detection of breast cancer in Nord-Trøndelag County, Norway [14]. Through linkage with data from the Cancer Registry of Norway, these women were followed for breast cancer occurrence. Between 1961 and 2008, 1393 new breast cancers were registered. All tumours were reclassified according to histological type and grade [12, 15]. Tissue microarray (TMA) blocks were made using the Tissue Arrayer Mini-Core with TMA Designer2 software (Alphelys). Three 1-mm-in-diameter tissue cores from the periphery of the FFPE primary tumours and lymph node metastases were transferred to TMA recipient blocks. TMA sections (4 μm) were cut and stained, and the tumours were reclassified into molecular subtypes. Human epidermal growth factor receptor 2 (HER2) was assessed using both CISH and IHC. Tumours with *HER2*/chromosome enumeration probe 17 (CEP17) ratio ≥ 2 were defined as HER2 positive. When CISH was unsuccessful, tumours with intense membranous staining (IHC 3+) in > 10% of tumour cells were considered HER2 positive.

Of the 1393 tumours, 909 were successfully reclassified into molecular subtypes [12]:Luminal A (ER and/or PR+ , HER2−, Ki67 < 15%), Luminal B (HER2−) (ER+ and/or PR + , HER2−, Ki67 ≥ 15%), Luminal B (HER2+) (ER+ and/or PR+ , HER2+), HER2 type (ER− and PR− , HER2+), 5 negative phenotype (5NP; ER−, PR−, HER2−, CK5− and EGFR−) and Basal phenotype (BP; ER− , PR− , HER2 − , CK5+ and/or EGFR+) (Supplementary Fig. 1). From the time of diagnosis (baseline), patients were followed until death from breast cancer, death from other causes or until December 31st, 2015. Individual information about adjuvant treatment is unavailable. However, due to age and/or time of diagnosis, few would have received chemotherapy. Some would have been treated with antihormonal treatment, but none qualified for trastuzumab. In the present study, TMAs containing cores from tumours diagnosed mainly in the 1980s or later (*n* = 636) were included. Of these, 46 were excluded due to unsuccessful FISH (*n* = 30) or insufficient amounts of tumour tissue (*n* = 16). Thus, 590 cases were suitable for *MRPS23* and CEP17 copy number assessment. Of these, 192 had lymph node metastases, and lymph node tissue from 150 was available in TMAs. Due to unsuccessful FISH (*n* = 5) or insufficient amounts of tissue (*n* = 1), six were excluded. Hence, lymph node metastases from 144 cases were included.

#### Cohort 2 METABRIC

The METABRIC dataset includes a discovery dataset (*n* = 997), and a validation dataset (*n* = 995). The cohorts have previously been described in detail [13]. In the present study, 1971 cases had available follow-up data and *MRPS23* gene expression data from all primary breast tumours. Tumours of basal-like (*n* = 329), normal-like (*n* = 202) and unknown subtype (*n* = 6) were excluded from our analysis [3, 16, 17]. Thus, 1434 tumours were included. Patients with ER positive and/or lymph node negative tumours had not received chemotherapy, whereas patients with ER negative and lymph node positive tumours did [13]. None of the HER2+ patients were treated with trastuzumab. To assess possible associations between gene expression levels and prognosis, cases were separated into quartiles. Prognosis for each quartile was analysed separately, and for dichotomization of gene expression values, patients with gene expression levels in the upper quartile were compared to all other cases.

#### *MRPS23* FISH cohort 1

FISH was done according to the manufacturer’s guidelines using Dako Histology FISH Accessory Kit K 579911. After de-waxing and rehydration, TMA slides were boiled in a microwave oven (10 min) in Pre-Treatment Solution, cooled (15 min) and washed in Wash Buffer (2 × 3 min). Protein digestion was performed with Pepsin Solution at 37 °C (30 min), and then washed in Wash buffer (2 × 3 min). Dehydration was done in ethanol (70, 80 and 95%) for 2 min at each concentration, and the slides were then air dried at room temperature for 15 min. FISH-custom probes for *MRPS23* (3 μL, Empire Genomics) and CEP17 (1 μL, Abbott/VYSIS) were mixed with hybridization buffer (9 μL, Empire Genomics) and applied to TMA slides. Coverslips were applied and sealed with coverslip sealant (Dako). Denaturation was performed at 83 °C (3 min) followed by hybridization at 37 °C overnight in a DAKO Hybridizer. Post hybridization, TMA slides were rinsed in 0.4xSSC/0.3%NP-40 at 72 °C (2 min), and in 2xSSC/0.1%NP-40 at RT (15 s). Slides were air dried at 37 °C (15 min). DAPI (15 μL, VYSIS. Abbott no 06J50-001) was applied and the slides were coverslipped.

*MRPS23* and CEP17 copy numbers were counted using a fluorescence microscope (Nikon Eclipse 90i). All available tissue cylinders from each case were examined, and *MRPS23* and CEP17 copy number in 20 well-preserved, non-overlapping tumour cell nuclei were recorded. Mean copy number of *MRPS23*/tumour cell and *MRPS23*/CEP17 ratio were estimated for each case. To assess the impact of mean *MRPS23* copy number, cases were divided into three categories based on recent HER2 guidelines [18]: mean *MRPS23* copy number < 4; mean ≥ 4 < 6 and mean ≥ 6. To estimate the impact of gene/centromere ratio, cases were divided into two categories: *MRPS23*/CEP17 < 2 and *MRPS23*/CEP17 ≥ 2. Finally, *MRPS23* amplification (*MRPS23*+) was defined as mean *MRPS23* copy number ≥ 6 and/or *MRPS23*/CEP17 ratio ≥ 2. Cases with *MRPS23* copy number < 6 and *MRPS23*/CEP17 ratio < 2 were defined as non-amplified (*MRPS23−*). To study the prognostic value of HER2 status and *MRPS23* status combined, cases were divided into four groups: *MRPS23−/*HER2−; *MRPS23−/*HER2+ ; *MRPS23* + */*HER2− and *MRPS23*+*/*HER2+. The REMARK criteria for tumour marker reporting were followed [19].

### Statistical analyses

Pearson chi square tests were used to compare *MRPS23* copy number status and *MRPS23* gene expression levels in the primary tumours across patient and tumour characteristics. To compare copy number status in the primary tumours and their corresponding lymph node metastases, paired analyses were performed using McNemar’s and marginal homogeneity test.

For each category of *MRPS23* copy number status and *MRPS23/*HER2 status in primary tumours, and for the *MRPS23* gene expression categories, we estimated cumulative incidence of death from breast cancer, with death from other causes as a competing event. We used Gray’s test to compare equality of cumulative incidence curves. Cox proportional hazard models were used to estimate hazard ratios (HR) of breast cancer death with 95% confidence intervals (CI). In the Cox regression analyses, patients were censored at time of death from other causes. Where applicable, adjustments were made for age at baseline (≤ 49, 50–59, 60–64, 65–69, 70–74, ≥ 75), histological grade (I–III), stage (I–IV), Ki67 status (< / ≥ 15%) and molecular subtype. We found no clear violations of proportionality in log-minus-log plots. Linear regression analyses were used for comparison of *MRPS23* expression levels between different molecular subtypes.

All statistical tests were two-sided, and statistical significance was assessed at 5% level. *p*-values between 5 and 10% were regarded borderline significant. All statistical analyses were done using STATA version 15.1 (Stata Corp., College Station, TX, USA).

## Results

### Cohort 1

Mean age at diagnosis was 75.6 (SD 8.6, range 41–96) years and mean follow-up after diagnosis was 8.9 (SD 7.2) years. By the end of follow-up, 217 (37%) patients had died from breast cancer, and 318 (54%) had died from other causes (Table 1).

**Table 1:** Characteristics of cohort 1

	Total study population	Categories defined by mean *MRPS23*				Categories defined by *MRPS23*/CEP17 ratio			Categories defined by amplification status		
		< 4	≥ 4 to < 6	≥ 6	*p* value (*χ*^2^)	< 2	≥ 2	*p* value (*χ*^2^)	*MRPS23*/CEP17 < 2 and mean *MRPS23* < 6	*MRPS23*/CEP17 ≥ 2 and/or mean *MRPS23* ≥ 6	*p* value (*χ*^2^)
*N* (%)	590 (100)	540 (92)	29 (5)	21 (4)		549 (93)	41 (7)		545 (92)	45 (8)	
Mean age at diagnosis, years (SD)	75.6 (8.6)	75.6 (8.5)	76.3 (7.7)	74.4 (11.2)		75.6 (8.5)	74.5 (9.5)		75.6 (8.5)	74.4 (9.4)	
Mean follow-up, years (SD)	8.9 (7.2)	8.8 (7.2)	10.7 (8.1)	7.6 (6.6)		8.9 (7.2)	8.7 (7.6)		8.9 (7.2)	8.6 (7.3)	
Deaths from BC (%)	217 (37)	196 (36)	11 (38)	10 (48)		199 (36)	18 (44)		198 (36)	19 (42)	
Deaths from other causes (%)	318 (54)	292 (54)	15 (52)	11 (52)		297 (51)	21 (51)		294 (54)	24 (53)	
Histologic grade (%)											
I	70 (12)	69 (13)	0	1 (5)	< 0.001	69 (13)	1 (2)	0.006	69 (13)	1 (2)	0.001
II	327 (55)	308 (57)	12 (41)	7 (33)		309 (56)	18 (44)		308 (56)	19 (42)	
III	193 (33)	163 (30)	17 (58)	13 (61)		171 (31)	22 (54)		168 (31)	25 (56)	
Unknown											
Lymph node metastasis (%)											
Yes	192 (33)	173 (32)	13 (45)	6 (29)	0.691	176 (32)	16 (40)	0.283	175 (32)	17 (38)	0.458
No	245 (41)	226 (42)	12 (42)	7 (33)		231 (42)	14 (34)		228 (42)	17 (38)	
Unknown histology	153 (26)	141 (26)	4 (14)	8 (38)		142 (26)	11 (27)		142 (26)	11 (24)	
Tumour size (%)											
≤ 2 cm	282 (48)	260 (48)	15 (52)	7 (33)	0.40	262 (48)	20 (49)	0.514	261 (48)	21 (47)	0.264
> 2 cm, ≤ 5 cm	96 (16)	87 (16)	4 (14)	5 (24)		90 (16)	6 (15)		89 (16)	7 (16)	
> 5 cm	10 (2)	10 (2)	0	0		10 (2)	0		10 (2)	0	
Uncertain, but > 2 cm	74 (12)	63 (12)	6 (21)	5 (24)		66 (12)	8 (19)		64 (12)	10 (22)	
Uncertain	128 (22)	120 (22)	4 (14)	4 (19)		121 (22)	7 (17)		121 (22)	7 (16)	
Stage (%)											
I	286 (49)	267 (49)	9 (31)	10 (48)	0.024	269 (49)	17 (41)	0.157	267 (49)	19 (42)	0.182
II	239 (41)	214 (40)	17 (59)	8 (38)		220 (40)	19 (46)		219 (40)	20 (44)	
III	35 (6)	33 (6)	1 (3)	1 (5)		33 (6)	2 (5)		33 (6)	2 (4)	
IV	28 (5)	25 (5)	2 (7)	1 (5)		26 (5)	2 (5)		25 (5)	3 (7)	
Unknown	2	1 (0.2)	0	1 (5)		1 (0)	1 (2)		1 (0.2)	1 (2)	
Molecular subtype (%)											
Luminal A	309 (52)	295 (55)	11 (38)	3 (14)	< 0.001	301 (55)	8 (20)	< 0.001	301 (55)	8 (18)	< 0.001
Luminal B (HER2−)	148 (25)	131 (24)	9 (31)	8 (38)		132 (24)	16 (39)		130 (24)	18 (40)	
Luminal B (HER2+)	44 (7)	34 (6)	4 (14)	6 (29)		31 (6)	13 (32)		31 (6)	13 (29)	
HER2 type	33 (6)	26 (5)	3 (10)	4 (19)		29 (5)	4 (10)		27 (5)	6 (13)	
5NP	14 (2)	14 (3)	0	0		14 (3)	0		14 (3)	0	
BP	42 (7)	40 (7)	2 (7)	0		42 (8)	0		42 (8)	0	
Histological subtype (%)											
Ductal carcinoma	409 (69)	373 (69)	24 (83)	12 (57)	0.695	380 (69)	29 (71)	0.690	377 (69)	32 (71)	0.840
Lobular carcinoma	78 (13)	75 (14)	0	3 (14)		74 (13)	4 (10)		74 (14)	4 (9)	
Tubular carcinoma	3 (1)	3 (1)	0	0		3 (1)	0		3 (1)	0	
Mucinous carcinoma	30 (5)	28 (5)	1 (3)	1 (5)		28 (5)	2 (5)		28 (5)	2 (4)	
Medullary carcinoma	14 (2)	12 (2)	1 (3)	1 (5)		14 (3)	0		13 (2)	1 (2)	
Papillary carcinoma	27 (5)	23 (4)	2 (7)	2 (10)		24 (4)	3 (7)		24 (2)	3 (7)	
Metaplastic	8 (1)	8 (1)	0	0		8 (1)	0		8 (1)	0	
Other	21 (4)	18 (3)	1 (3)	2 (10)		18 (3)	3 (7)		18 (3)	3 (7)	
Ki67 high/low (%)											
Ki67 < 15%	349 (59)	330 (61)	13 (45)	6 (29)	0.003	335 (61)	14 (34)	0.001	334 (61)	15 (33)	< 0.001
Ki67 ≥ 15%	241 (41)	210 (39)	16 (55)	15 (71)		214 (39)	27 (66)		211 (39)	30 (66)	
Mitoses/10 HPF, median (IQR)	5 (1.12)	5 (1.12)	9 (3.17)	10 (5.16)		5 (1.12)	8 (3.16)		5 (1.11)	8 (4.17)	
Mitoses/10 HPF, quartiles (%)											
≤ 1	154 (26)	148 (27)	5 (17)	1 (5)	0.146	148 (27)	6 (15)	0.197	148 (27)	6 (13)	0.058
> 1, ≥ 5	158 (27)	146 (27)	6 (21)	6 (29)		148 (27)	10 (24)		147 (27)	11 (24)	
> 5, ≤ 12	140 (24)	126 (23)	8 (28)	6 (29)		129 (24)	11 (27)		129 (24)	11 (24)	
> 12	138 (23)	120 (22)	10 (34)	8 (38)		124 (23)	14 (34)		121 (22)	17 (38)	

*N* number of patients, *SD* standard deviation, *BC* breast cancer, *HER2* Human epidermal growth factor receptor 2, *5NP* five negative phenotype, *BP* basal phenotype, *HPF* High- power filed, *IQR* inter quartile range

### *MRPS23* copy number status in primary tumours

**Fig. 1:**
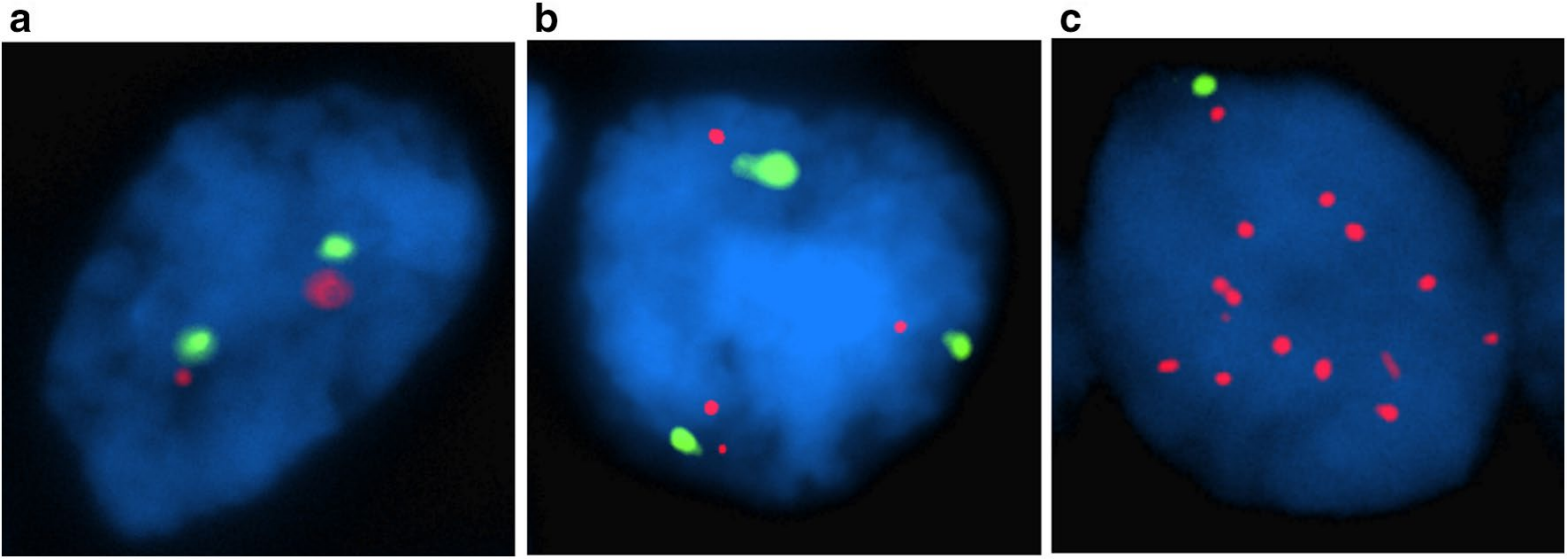
Breast cancer cell nucleus with **a** two copies of *MRPS23* and CEP17, **b** copy number increase of both *MRPS23* and CEP17 and **c** copy number increase of *MRPS23* without corresponding increase of CEP17

**Fig. 2:**
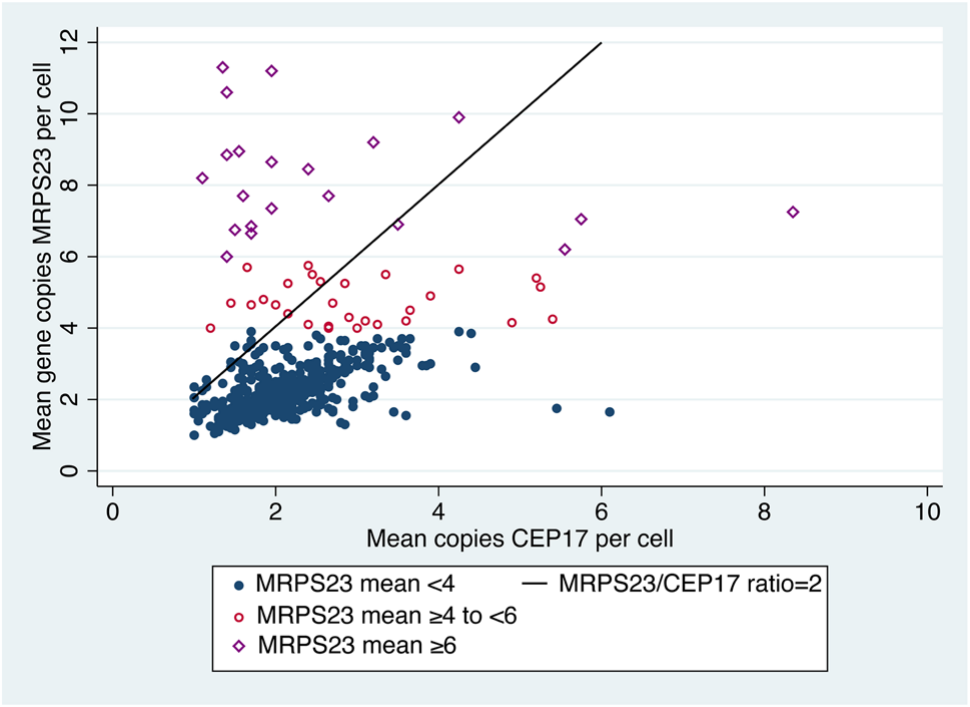
Scatter plot of *MRPS23* and CEP17 copy number in 590 breast cancer tumours

When *MRPS23* and CEP17 copy number alterations were present, a homogenous pattern was seen, with alterations in the majority of tumour cells. Three different phenotypes were seen, tumours without copy number alterations; tumours with *MRPS23* and CEP17 copy number increase and tumours with *MRPS23* copy number increase only (Fig. 1). In total, 29 of the primary tumours (5%) had mean *MRPS23* copy number ≥ 4 < 6, 21 (4%) had mean copy number ≥ 6 and 41 (7%) had *MRPS23/*CEP17 ratio ≥ 2 (Table 1, Fig. 2). A total of 45 tumours (8%) were amplified (mean *MRPS23* ≥ 6 and/or *MRPS23*/CEP17 ≥ 2). Among cases with mean *MRPS23* ≥ 6, four cases had *MRPS23*/CEP17 ratio < 2. Of cases with *MRPS23*/CEP17 ratio ≥ 2, 24 had mean *MRPS23* < 6.

*MRPS23* copy number increase (mean ≥ 4) was found within all molecular subtypes except the 5NP (Table 1). Amplifications were found in all molecular subtypes except the 5NP and the BP. Amplifications were seen in 30% of Luminal B (HER2+)*,* 18% of HER2 type, 12% of Luminal B (HER2*−*)*,* and 3% of Luminal A. Of the *MRPS23* amplified tumours, 19 (42%) were HER2+ , compared to 58 (11%) of non-amplified tumours.

### Copy number status in lymph node metastases

In total, 144 cases were examined for *MRPS23* copy number status in their lymph node metastases (Table 2). There were no significant changes in *MRPS23* copy number status in the lymph node metastases compared to the corresponding primary tumours. Among the pairs of primary tumours and lymph node metastases, 14 (10%) primary tumours were classified as *MRPS23*+ , and in 10 of these (71%), the corresponding lymph node metastases were also *MRPS23*+ . *MRPS23* amplification was also identified in the lymph node metastases of two *MRPS23*-tumours.

**Table 2:** *MRPS23* status in primary tumours and lymph node metastases according to *MRPS23*/CEP17 ratio, mean *MRPS23* and amplification status

	Mean *MRPS23*/tumour cell, primary tumours				Marginal homogeneity test
	< 4	≥ 4, < 6	≥ 6	Total	
Mean *MRPS23*/tumour cell, lymph nodes					
< 4	125 (97)	4 (40)	1 (20)	130	*p* = 0.637
≥ 4, < 6	3 (2)	5 (50)	2 (40)	10	
≥ 6	1 (1)	1 (10)	2 (40)	4	
Total	129	10	5	144	

	*MRPS23*/CEP17 ratio, primary tumours			
	< 2	≥ 2	Total	McNemar test
*MRPS23*/CEP17 ratio, lymph nodes				
< 2	130 (99)	3 (23)	133	*p* = 0.625
≥ 2	1 (1)	10 (76)	11	
Total	131	13	144	

	Amplification status, primary tumours			
	*MRPS23*−	*MRPS23*+	Total	McNemar test
Amplification status, lymph nodes				
*MRPS23*−^a^	128 (98)	4 (29)	132	*p* = 0.688
*MRPS23*+^b^	2 (2)	10 (71)	12	
Total	130	14	144	

^a^*MRPS23*/CEP17 < 2 and mean *MRPS23* < 6

^b^*MRPS23*/CEP17 ≥ 2 and/or mean *MRPS23* ≥ 6

### *MRPS23* copy number status and proliferation

Of the *MRPS23* amplified tumours, 66% had high Ki67 (≥ 15%), compared to 39% of the non-amplified tumours (Table 1). Mitotic counts were also higher in amplified tumours (borderline significance). Of the *MRPS23* amplified tumours, 56% were grade III, compared to 31% of the non-amplified tumours. An association between *MRPS23* copy number increase and high Ki67, and high histological grade was also found when *MRPS23* copy number status was defined by *MRPS23* mean and *MRPS23*/CEP17 ratio (Table 1). In the lymph node metastases, *MRPS23* amplification was associated with high Ki67 (borderline significance, Table 3).

**Table 3:** *MRPS23* amplification status and Ki67 levels in lymph nodes

	*MRPS23* amplification status, lymph node			
	*MRPS23−*^a^	*MRPS23*+^b^	Total	*χ*^2^
Ki67, lymph node (%)				
Ki67 < 15%	69 (53%)	3 (25%)	72	*p* = 0.07
Ki67 > 15%	62 (47%)	9 (75%)	71	
Total	131	12	143	

^a^*MRPS23*/CEP17 < 2 and mean *MRPS23* < 6

^b^*MRPS23*/CEP17 ≥ 2 and/or mean *MRPS23* ≥ 6

### *MRPS23* copy number status and prognosis

#### Mean *MRPS23*

**Fig. 3:**
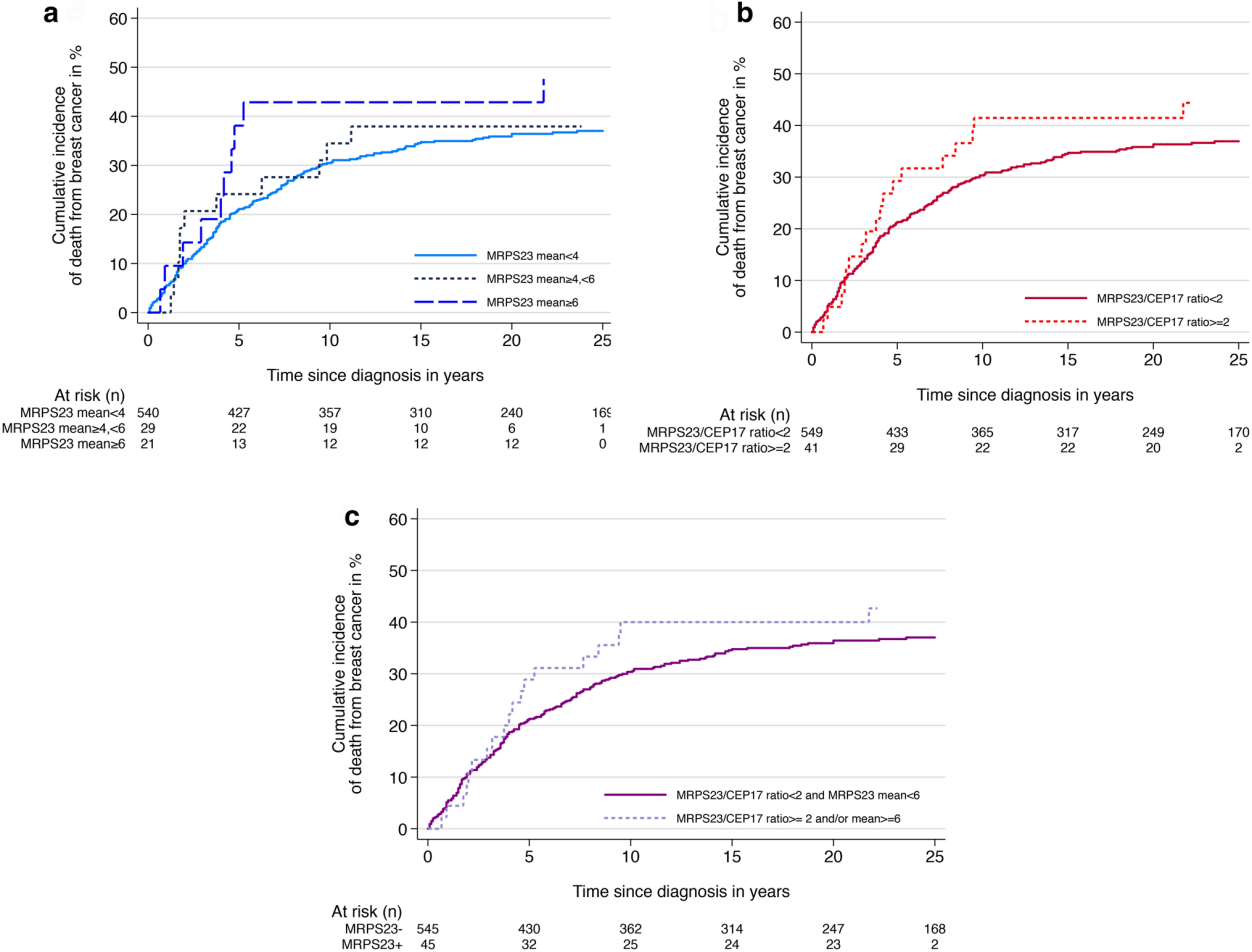
Cumulative incidence of death from breast cancer according to *MRPS23* copy number status based on **a** mean *MRPS23* copy number (*p* = 0.47), **b***MRPS23*/CEP17 ratio (*p* = 0.29) and **c***MRPS23* amplification status (*p* = 0.39)

The cumulative risk of death from breast cancer for cases with no copy number increase was 35% (95% CI 31–39) 10 years after diagnosis (Table 4; Fig. 3a). The corresponding risks for cases with mean copy number ≥ 4 < 6 and mean ≥ 6 was 34% (95% CI 20–55), and 43% (95% CI 25–66), respectively. In the Cox regression analysis, there were no clear differences in the rates of death between categories. Adjustments for age, stage, grade, Ki67 or HER2 status did not influence the results.

**Table 4:** Absolute and relative risk of death from breast cancer according to mean *MRPS23*/tumour cell, *MRPS23*/CEP17 ratio and amplification status

	Mean *MRPS23*/tumour cell, primary tumour			*MRPS23*/CEP17 ratio, primary tumour		Amplification status, primary tumours	
	< 4	≥ 4, < 6	≥ 6	< 2	≥ 2	*MRPS23*−^a^	*MRPS23*+^b^
Cum. risk after 5 years (%) (95% CI)	21 (18–24)	24 (12–44)	38 (21–62)	21 (18–25)	29 (18–46)	21 (18–25)	29 (18–44)
Cum. risk after 10 years (%) (95% CI)	35 (31–39)	34 (20–55)	43 (25–66)	30 (27–34)	41 (30–58)	30 (27–34)	40 (27–56)
HR, unadjusted (95% CI)	1.0	0.9 (0.5–1.7)	1.5 (0.8–2.8)	1.0	1.2 (0.8–2.0)	1.0	1.2 (0.7–1.9)
HR adjusted for age (95% CI)	1.0	0.9 (0.5–1.7)	1.5 (0.8–2.8)	1.0	1.3 (0.8–2.1)	1.0	1.2 (0.7–1.9)
HR adjusted for stage (95% CI)	1.0	0.8 (0.4–1.5)	1.2 (0.6–2.4)	1.0	1.2 (0.7–1.9)	1.0	1.0 (0.6–1.7)
HR adjusted for grade (95% CI)	1.0	0.7 (0.4–1.4)	1.3 (0.7–2.4)	1.0	1.1 (0.7–1.7)	1.0	1.0 (0.6–1.6)
HR adjusted for Ki67 (95% CI)	1.0	0.7 (0.4–1.4)	1.1 (0.6–2.2)	1.0	1.0 (0.6–1.6)	1.0	1.0 (0.6–1.6)
HR adjusted for HER2 (95% CI)	1.0	0.9 (0.5–1.6)	1.2 (0.6–2.3)	1.0	1.0 (0.6–1.7)	1.0	1.0 (0.6–1.6)

*CI* confidence interval, *HR* hazard ratio

^a^*MRPS23*/CEP17 < 2 and mean *MRPS23* < 6

^b^*MRPS23*/CEP17 ≥ 2 and/or mean *MRPS23* ≥ 6

#### *MRPS23*/CEP17 ratio

After 10 years of follow-up, cases with *MRPS23*/CEP17 ratio < 2 had a cumulative risk of death from breast cancer of 30% (95% CI 27–34) (Table 4; Fig. 3b), whereas cases with ratio ≥ 2 had a corresponding risk of 41% (95% CI 30–58). When comparing rates of death from breast cancer, there was no clear difference between the two categories (HR 1.2, 95% CI 0.8–2.0). Adjusting for age, stage, grade, Ki67 or HER2 status did not influence the results.

#### *MRPS23* amplification status

After 10 years of follow-up, patients with *MRPS23* amplified tumours had 40% (95% CI 27–56) cumulative risk of death from breast cancer, compared to 30% (95% CI 27–34) for patients without amplification (Table 4; Fig. 3c). The rates of death from breast cancer were similar for cases with and without amplification (HR 1.2, 95% CI 0.7–1.9). Separate adjustments for age, grade, histological grade, stage and HER2 status did not influence the results. Analysis of prognosis was also done for Luminal A cases separately, and for all luminal subtypes combined. In these analyses, no clear differences in prognosis between *MRPS23* amplified and non-amplified cases were seen (data not shown).

#### *MRPS23/*HER2− status

**Fig. 4:**
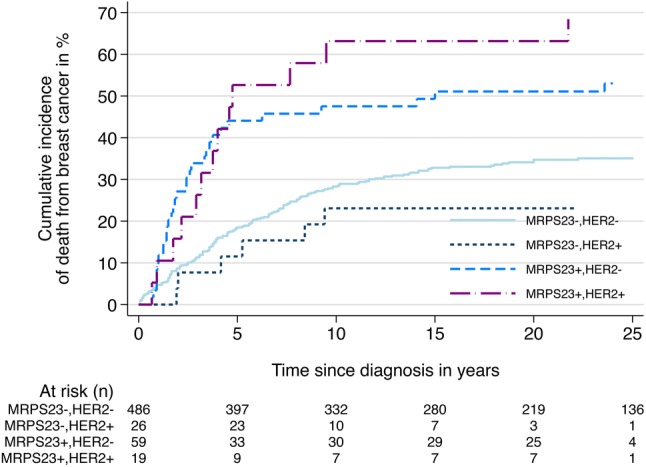
Cumulative incidence of death from breast cancer according to *MRPS23* amplification status and HER2 status (*p* < 0.01)

When tumours were reclassified into four categories based on *MRPS2*3 and HER2 status, the highest risk of death was found in the *MRPS23*+/HER2+ subtype (Table 5; Fig. 4). After 10 years of follow-up, patients with the *MRPS23*+/HER2+ subtype had a cumulative risk of death from breast cancer of 63% (95% CI 42–83). The corresponding risk for the *MRPS23*−/HER2+ subtype was 47% (95% CI 36–61). The lowest risk of death was found in the *MRPS23*+ /HER2- subtype (23%, 95% CI 11–44). There were no clear differences in the rate of death from breast cancer between the *MRPS23*−/HER2+ and *MRPS23*+ /HER2+ subtypes (HR 1.3, 95% CI 0.7–2.6). Separate analysis of prognosis was also done for all luminal cases, with similar results as for all cases combined (data not shown).

**Table 5:** Absolute and relative risk of dying from breast cancer according to HER2 status and amplification of *MRPS23*

	*MRPS23*−^a^/HER2−	*MRPS23−/*HER2+^c^	*MRPS23*+^b^/HER2−	*MRPS23*+/HER2+
Cum. risk after 5 years (%) (95% CI)	18 (15–22)	44 (32–58)	12 (4–32)	53 (33–76)
Cum. risk after 10 years (%) (95% CI)	28 (25–32)	47 (36–61)	23 (11–44)	63 (42–83)
HR, unadjusted (95% CI)	1.0	1.6 (1.1–2.4)	0.6 (0.3–1.4)	2.6 (1.5–4.5)
HR adjusted for age (95% CI)	1.0	1.7 (1.1–2.5)	0.6 (0.3–1.4)	2.6 (1.5–4.8)
HR adjusted for grade (95% CI)	1.0	1.2 (0.8–1.8)	0.5 (0.2–1.1)	2.3 (1.3–4.0)
HR adjusted for stage (95% CI)	1.0	1.9 (1.3–2.8)	0.6 (0.3–1.4)	1.8 (1.0–3.3)
HR adjusted for Ki67 (95% CI)	1.0	1.3 (0.9–1.9)	0.5 (0.2–1.0)	2.2 (1.3–3.0)
HR adjusted for HER2 (95% CI)	1.0	1.6 (1.1–2.4)	0.6 (0.3–1.4)	2.6 (1.5–4.5)

*Cum. risk* cumulative risk, *CI* confidence interval, *HR* hazard ratio

^a^*MPR23− : MPRS23*/CEP17 ratio < 2 and *MRPS23* mean < 6

^b^*MRPS23* + : *MRPS23*/CEP17 ratio ≥ 2 and/or *MRPS23* mean ≥ 6

^c^*HER2*/CEP17 ratio ≥ 2 or intense membranous staining (IHC 3+) in > 10% of tumour cells

### Cohort 2

#### *MRPS23* expression according to molecular subtypes

In the METABRIC dataset, mean age at diagnosis was 61.1 (SD 12.4, range 22–96) years, and mean follow-up after diagnosis was 8.1 (SD 4.9) years. Characteristics of the study population are given in Table 6. By the end of follow-up, 506 (26%) patients had died from breast cancer, and 384 (20%) had died from other causes.

**Table 6:** Characteristics of Cohort 2 (METABRIC) (Normal-like and basal-like subtypes excluded from the analyses)

	Total study population				Discovery cohort	Validation cohort
	Total	Mean probe *MRPS23*		χ^2^		
		Quartile 1–3^a^	Quartile 4^b^			
*N* (%)	1434	1069	365		804 (56)	630 (44)
Mean age at diagnosis, years (SD)	63.2 (12.4)	63.6 (12.4)	62 (12.4)		62.3 (12.5)	64.3 (12.2)
Mean follow-up, years (SD)	8.3 (4.9)	8.5 (5)	7.8 (4.5)		8.1 (4.7)	8.5 (5.1)
Deaths from BC (%)	354 (25)	273 (25)	81 (22)		201 (25)	153 (24)
Deaths from other causes (%)	311 (22)	240 (22)	71 (19)		156 (19)	155 (25)
Histologic grade (%)						
I	139 (10)	116 (11)	23 (6)	< 0.001	61 (8)	78 (12)
II	635 (44)	504 (47)	131 (36)		375 (47)	260 (41)
III	598 (42)	392 (17)	206 (56)		368 (46)	230 (37)
Unknown	62 (4)	57 (5)	5 (1)		0	62 (10)
Lymph node metastasis (%)						
Yes	684 (48)	491 (46)	193 (53)	0.030	383 (48)	301 (48)
No	750 (52)	578 (54)	172 (47)		421 (52)	329 (52)
Tumour size (%)						
≤ 2 cm	613 (43)	472 (44)	141 (39)	0.170	351 (42)	262 (42)
> 2 cm, ≤ 5 cm	751 (52)	542 (51)	209 (57)		421 (52)	330 (53)
> 5 cm	69 (5)	54 (5)	15 (4)		32 (4)	37 (6)
Uncertain	1 (0)	1 (0)	0		0	1 (0)
Stage (%)						
I	104 (7)	84 (8)	20 (5)	0.075	0	104 (17)
II	177 (12)	141 (13)	36 (10)		0	177 (28)
III	27 (2)	20 (2)	7 (2)		0	27 (4)
IV	1 (0)	0	1 (0)		0	1 (0)
Unknown	1125 (78)	824 (77)	301 (82)		804 (100)	321 (50)
PAM50 subtype (%)						
Luminal A	709 (49)	606 (57)	103 (28)		445 (56)	255 (40)
Luminal B	488 (34)	276 (26)	212 (58)		266 (33)	222 (35)
HER2-type	237 (17)	187 (17)	50 (14)	< 0.001	84 (10)	153 (25)

*N* number of patients, *SD* standard deviation, *BC* breast cancer

^a^Mean probe *MRPS23* ≤ 8.31

^b^Mean probe *MRPS23* > 8.31

**Fig. 5:**
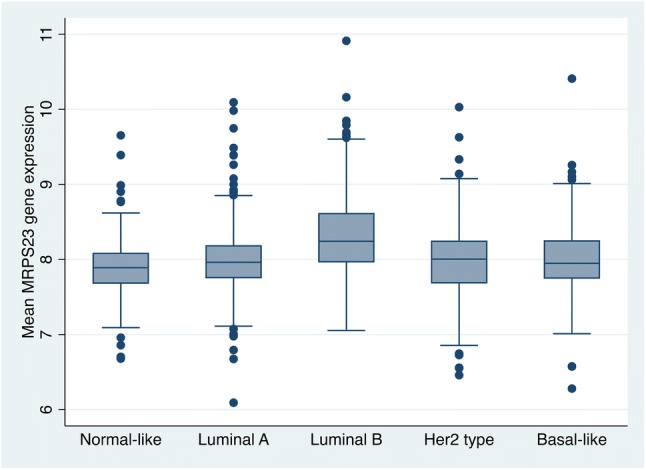
*MRPS23* gene expression according to molecular subtype in 1971 patients from the METABRIC dataset

High *MRPS23* expression levels were associated with the Luminal B subtype (Fig. 5).

**Fig. 6:**
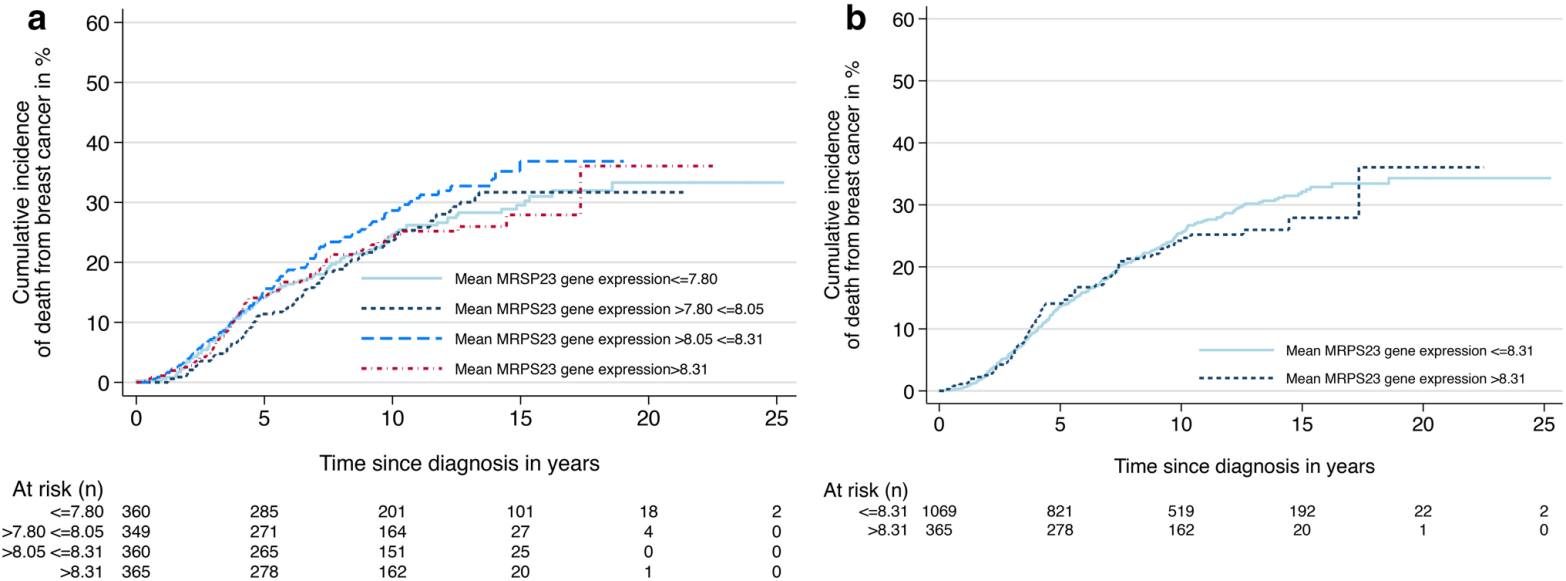
Cumulative incidence of death from breast cancer according to *MRPS23* gene expression divided into **a** quartiles 1–4 (*p* = 0.4), and **b** quartile 1–3 vs. quartile 4 (*p* = 0.6)

After 10 years of follow-up, cases with low *MRPS23* expression levels had a cumulative risk of death from breast cancer of 26% (95% CI 23–29%), compared to 24% (95% CI 20–30%) among cases with high *MRPS23* expression (Cut-off upper quartile; Fig. 6, Table 7). Comparing the rates of death from breast cancer, there were no significant differences between cases with gene expression levels in the upper quartile compared to the rest (HR 0.9, 95% CI 0.7–1.2, Fig. 6, Table 7). Similar results were obtained when analysis of prognosis was done for each quartile separately. Adjustments for age and histological grade did not influence the results.

**Table 7:** Absolute and relative risk of death from breast cancer according to gene expression levels of *MRPS23*, METABRIC data

	Mean probe MRPS23, quartiles					Mean probe MRPS23	
	Quartile 1^a^	Quartile 2^b^	Quartile 3^c^	Quartile 4^d^		Quartile 1–3	Quartile 4
Cum. risk after 5 years (%) (95% CI)	14 (11–18)	11 (8–15)	15 (12–20)	14 (11–18)		14 (12–16)	14 (11–18)
Cum. risk after 10 years (%) (95% CI)	25 (20–30)	23 (19–29)	29 (24–34)	24 (20–30)		26 (23–29)	24 (20–30)
HR, unadjusted (95% CI)	1	1.0 (0.7–1.3)	1.2 (0.9–1.6)	1.0 (0.7–1.3)		1	0.9 (0.7–1.2)
HR, adjusted for age (95% CI)	1	1.0 (0.7–1.4)	1.2 (0.9–1.6)	1.0 (0.7–1.4)		1	0.9 (0.7–1.2)
HR, adjusted for grade (95% CI)	1	1.0 (0.7–1.4)	1.2 (0.9–1.6)	0.9 (0.7–1.2)		1	0.8 (0.7–1.1)

*Cum. risk* cumulative risk, *CI* confidence interval, *HR* hazard ratio; Luminal A, Luminal B and HER2-type included in analyses

^a^Mean probe *MRPS23* ≤ 7.80

^b^Mean probe *MRPS23* > 7.80, ≤ 8.05

^c^Mean probe *MRPS23* > 8.05, ≤ 8.31

^d^Mean probe *MRPS23* > 8.31

## Discussion

We identified *MRPS23 *amplification in 8% of primary tumours and 9% of lymph node metastases in a large population of Norwegian breast cancer patients. The highest proportion of amplified cases was found within Luminal B (HER2+), HER2 type and Luminal B (HER2−) tumours. None of the amplified tumours were triple negative (5NP/BP). *MRPS23 *amplification was associated with high Ki67 and high histological grade. No clear association between *MRPS23* amplification and prognosis was seen. The proportion of HER2 positive cases was higher among *MRPS23* amplified cases, compared to non-amplified. *MRPS23*+/HER2+ had the poorest prognosis.

In the METABRIC dataset, Luminal B tumours had the highest level of *MRPS23* gene expression. We found no statistically significant associations between *MRPS23* expression levels and prognosis.

This study is based on a well-described cohort of breast cancer patients with long-term follow-up, and data from the METABRIC dataset. In the Norwegian cohort, the majority of patients have been followed until death [12]. Since relapse may occur even decades after the primary diagnosis, long-term follow-up is of particular value in breast cancer research. Molecular subtyping was performed in the same laboratory, using the same algorithm and antibodies in all cases [12]. Using FISH, gene copy number can be assessed while observing the morphology of the tumour, ensuring that only invasive tumour cells were examined.

*MRPS23* copy number in primary tumours and lymph node metastases was assessed in TMAs. In the primary tumours, tissue for TMAs was taken from the tumour periphery. Previous studies have shown good correlation between TMAs and corresponding whole sections [20, 21]. Nevertheless, TMAs represent a small portion of each tumour, and, while copy number changes were observed throughout the tissue in amplified cases, intra tumour heterogeneity may not be captured. It would therefore be of interest to validate our findings in a study of whole sections.

There are no established guidelines as to how *MRPS23* amplification should be defined. According to *HER2* ISH guidelines, both *HER2* copy number and *HER2*/CEP17-ratio are taken into consideration [18]. *MRPS23* and *HER2* are both located on the long arm of chromosome 17 [4, 5, 22], and we chose to define *MRPS23* amplification according to *HER2* ISH guidelines, including both mean *MRPS23* copy number and *MRPS23*/CEP17 ratio in our definition. Previous studies have shown a high frequency of abnormalities on chromosome 17, but rarely true polysomy [23]. The number of amplified cases was increased when including *MRPS23*/CEP17 ratio in addition to mean *MRPS23* in the definition of amplification. Hence, our definition may have led to overestimation of *MRPS23* amplified cases. Nevertheless, we found that 8% of tumours were *MRPS23* amplified, whereas 20% and 33% of the tumours were amplified in the two datasets included in Gatza et al. In the latter two cohorts, only luminal (defined as non-basal) cases were included. When excluding the BP and 5NP in our in-house cohort, the proportion of amplified cases was still 8%. In accordance with other studies we found that amplification of *MRPS23* was associated with higher proliferation [3, 11]. However, contrary to others, we found no clear associations between *MRPS23* copy number increase and a poorer prognosis [3]. Our study demonstrates the importance of validating biomarkers identified by high-throughput genomic analyses. Validation analyses of single biomarkers with FISH, performed in FFPE tissue, indicate the marker’s prognostic potential when assessed in a routine diagnostic setting.

*MRPS23* has previously been found to be amplified exclusively in highly proliferative luminal tumours [3]. In that study, PAM50 was used for molecular subtyping, and “luminal” was defined as all tumours that were not basal [3]. This definition of luminal was based on a study showing that breast tumours could be separated into two main groups, one group containing luminal and HER2-positive tumours and the other group comprising basal-like tumours [17]. In our study, cohort 1 was divided into six subtypes based on IHC and ISH. Although it has been shown that surrogate markers can be used for molecular subtyping [24–27], there is a discrepancy between molecular subtype defined by surrogate markers and subtypes defined by gene expression analyses [28, 29]. Nevertheless, similar to Gatza et al., we only found *MRPS23* amplified cases among the non-basal tumours.

Contrary to others [3], we found no clear associations between *MRPS23* amplification and prognosis in our cohort of Norwegian breast cancer patients. We used a different method for assessment of gene copy number in our study (FISH), and different methodologies could partly explain the divergent results. Furthermore, the number of amplified cases was low in our study population, and the results must be interpreted with caution. Patient age in Cohort 1 is high, and the majority of patients were not given modern breast cancer treatment either due to their age at diagnosis or time of diagnosis [12]. This enables us to follow the near-natural course of disease after surgery. It would, however, be interesting to perform a *MRPS23* FISH study in a cohort of younger patients treated according to current guidelines.

Interestingly, the *MRPS23*+ */*HER2+ tumours had the poorest prognosis. HER2 is recognized as an important prognostic marker in breast cancer, and interaction with *MRPS23* could potentially be of clinical importance. However, due to the low number of cases in some of the *MRPS23*/HER2 categories, the results should be interpreted with caution.

We found no correlation between *MRPS23* expression levels and prognosis in the METABRIC data set. In our analyses of prognosis, we excluded basal-like and normal-like cases [3, 16]. A correlation between *MRPS23* copy number status and gene expression has previously been found in the METABRIC dataset [3]. Since transcription is regulated by several mechanisms, good correlation between gene copy number and gene expression is infrequent [30–32]. Our analyses show that *MRPS23* expression levels were overlapping between the different molecular subtypes. Such overlap was also seen between the highly proliferative luminal tumours (Luminal B) and basal-like tumours, the latter shown to be non-amplified. This could possibly be due to other *MRPS23* up-regulating mechanisms, and potentially explain the lack of correlation between gene expression levels and prognosis in the METABRIC dataset.

## Conclusion

Using FISH on a large cohort of breast cancer patients we found that *MRPS23* amplification is associated with higher tumour cell proliferation. Amplifications were only found in luminal and HER2 type tumours. We found no association between *MRPS23* amplification and prognosis. In the METABRIC dataset, gene expression levels were highest in Luminal B tumours. There was no correlation between *MRPS23* expression and prognosis.

## Electronic supplementary material

Below is the link to the electronic supplementary material.

Supplementary file1 Algorithm for molecular subtyping (Engstrom et al [12]). Abbreviations: ER = estrogen receptor, PR = progesterone receptor, HER2 = human epidermal growth factor receptor 2, CK5 = cytokeratin 5, EGFR = epidermal growth factor (TIFF 3039 kb)
